# What is the hemodynamic effect of the Woven EndoBridge? An in vivo quantification using time-density curve analysis

**DOI:** 10.1007/s00234-020-02390-3

**Published:** 2020-03-13

**Authors:** Philipp Gölitz, Hannes Luecking, Philip Hoelter, Frauke Knossalla, Arnd Doerfler

**Affiliations:** 1 Department of Neuroradiology, University of Erlangen- Nuremberg, Schwabachanlage 6, 91054 Erlangen, Germany; 2 Department of Neurology, University of Erlangen- Nuremberg, Erlangen, Germany

**Keywords:** Aneurysm, Hemodynamics, Intrasaccular flow diverter, Digital subtraction angiography, Time-density curve, Image postprocessing

## Abstract

**Purpose:**

Using the Woven EndoBridge (WEB) for aneurysm treatment has emerged as endovascular approach aiming for flow disruption in aneurysm sac. Since quantifiable data confirming the hemodynamic effect are lacking, we investigated in vivo aneurysmal flow alterations using time-density curve (TDC) analysis. Additionally, we evaluated whether flow parameters could be identified as independent factor to predict aneurysm occlusion.

**Methods:**

Forty cerebral aneurysm patients treated with WEB were enrolled. Pre- and postinterventional digital subtraction angiography series were postprocessed and TDCs generated. TDCs were quantified calculating the parameters aneurysmal inflow velocity, outflow velocity, mean flow velocity, and relative time-to-peak (rTTP) of aneurysm filling. Pre- and postinterventional values were compared and related to occlusion rate.

**Results:**

WEB implanting induced highly significant rTTP prolongation by 52% (*p* = 0.001) and highly significant decrease of aneurysmal inflow, outflow, and mean flow velocity (*p* < 0.001). While outflow velocity was reduced by 49%, inflow velocity was reduced by 33% only. No statistically significant difference between the occluded and the non-occluded group was observed. No flow parameter reached significance level concerning predicting aneurysm occlusion.

**Conclusion:**

Flow quantification confirms a significant flow-disrupting effect of WEB reducing more the outflow than the inflow velocity. In our small cohort, no flow parameter reached statistical significance to show predictive value regarding complete aneurysm occlusion. The hemodynamic effect of WEB is on comparable level to flow-diverting stents meaning that aneurysm closure can be delayed. In case of only slight inflow changes and high aneurysmal hemodynamic stress, some aneurysms might not be adequately protected in the short term.

## Introduction

Flow disruption using an intrasaccular flow diverter (Woven EndoBridge, WEB, Sequent Medical, Aliso Viejo, CA, USA) has been established as an innovative endovascular concept for the treatment of especially wide-neck aneurysms. Treatment intention is to disrupt the blood flow at the level of the aneurysm ostium and induce aneurysmal thrombosis. The WEB consists of a dense nitinol mesh and has been progressively developed since 2010 from a dual-layer version (WEB DL) [1] to single-layer versions (WEB SL and WEB SLS (single-layer spherical)) [2]. Actually, the new fifth generation of the WEB (WEB 17) is available. Several studies have demonstrated the safety and efficiency of the WEB [3–9]. But less is known about the precise intraaneurysmal flow alterations induced by the WEB and its real hemodynamic efficiency. In the first presentation of the WEB, the intraaneurysmal flow modulation after implantation was assessed only by visual analysis of intraaneurysmal contrast stagnation on digital subtraction angiography (DSA) series based on a scale not previously validated [10]. Alternatively, visual assessment of DSA series according to the Raymond scale [11], the Leeds WEB aneurysm occlusion scale [12], or the WEB occlusion scale [13] has been described, but quantifiable in vivo data are lacking. Furthermore, aneurysm recurrence after WEB implantation can occur [14] and it is not yet fully understood which aneurysms are more likely to recanalize and which flow parameters might play a role.

Objective of our study was to evaluate and quantify in vivo the intraaneurysmal hemodynamic changes induced by WEB deployment using time-density curve (TDC) analysis. Moreover, we aimed to identify flow parameters as independent factor that might allow to predict complete aneurysm occlusion.

## Materials and methods

Forty consecutive aneurysm patients (twenty-one women and nineteen men, aged 38–74 years, 57 median years) treated with WEB between March 2016 and September 2018 were retrospectively enrolled in this study in accordance with the guidelines of our institution and after obtaining written informed consent, if angiographic follow-up after 6 months was available. Except nine, the aneurysms were unruptured. The mean aneurysm dimensions were height of 5 (± 2.2) mm, width of 5 (± 1.7) mm, dome-to-neck-ratio of 1.4 (± 0.2), and aspect ratio of 1.4 (± 0.4). The aneurysms were located as follows: five at the carotid terminus (13%), fifteen at the middle cerebral artery bifurcation (38%), twelve at the anterior communicating artery (30%), one at the posterior cerebral artery (3%), four at the basilar artery tip (10%), and three at the M2 segment of the middle cerebral artery (8%). In all cases, the WEB SL was used for aneurysm treatment besides one case in which a WEB SLS was used.

In case of an unruptured aneurysm, dual antiplatelet therapy (aspirin, clopidogrel) was initiated 4 days before treatment and heparin was applied during the procedure. After WEB deployment, clopidogrel was stopped and aspirin was continued for 4 weeks. In case of a ruptured aneurysm, aspirin and heparin were applied during intervention and aspirin was continued for 4 weeks. Endovascular access was typically via a standard 6F guiding catheter, and the WEB was delivered through a VIA microcatheter (Sequent Medical, Aliso Viejo, CA, USA). WEB sizing was performed according to the manufacturer’s recommendation.

Aneurysmal occlusion status at angiographic follow-up after 6 months was assessed according to the five-grade Beaujon Occlusion Scale Score (BOSS classification) [15, 16]. Grade 0 (no residual flow in aneurysm/WEB) and 0′ (opacification of the proximal recess of WEB) were rated as complete occlusion, the other grades as non-occluded. Binary occlusion status was recorded.

### Image acquisition

DSA was performed under general anesthesia on a biplane flat-detector angiographic system (Artis zee Biplane System, Siemens AG, Healthineers). Vital parameters were monitored continuously and stabilized during intervention, especially during WEB deployment. Using standard angiographic methods (transfemoral route), a 6F guiding catheter was positioned in the petrous segment of the internal carotid artery (ICA) or the third segment of the vertebral artery (VA) in the case of aneurysms of posterior circulation. Pre- and postinterventional 2D DSA series were acquired at a rate of 4 frames per second. During intervention, catheter and table position remained unchanged. For image acquisition, 8 ml of contrast material (Imeron 300, Bracco Imaging) was injected manually by one experienced operator at a flow rate of about 4 ml/s.

### Image analysis and statistics

**Fig. 1:**
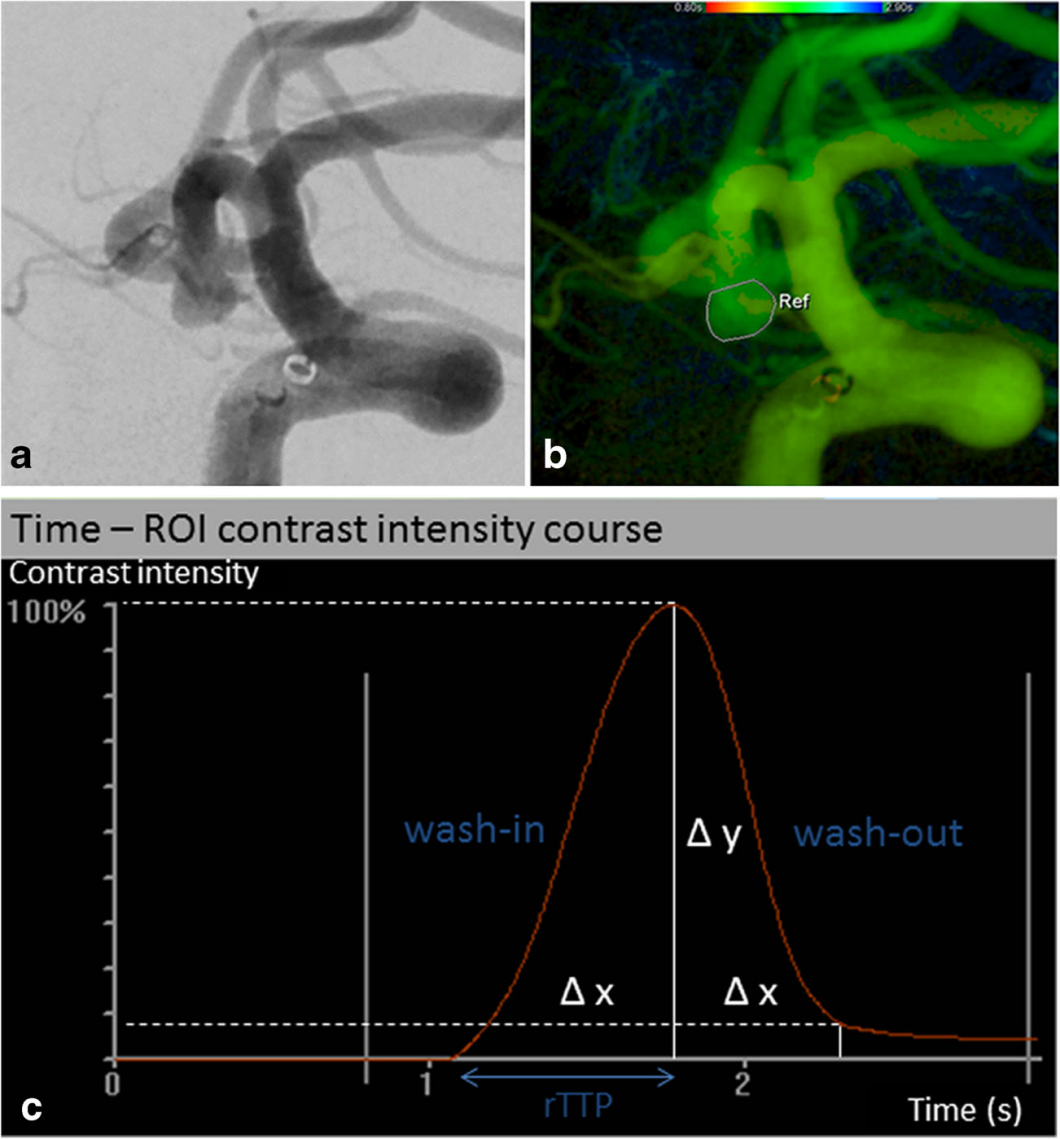

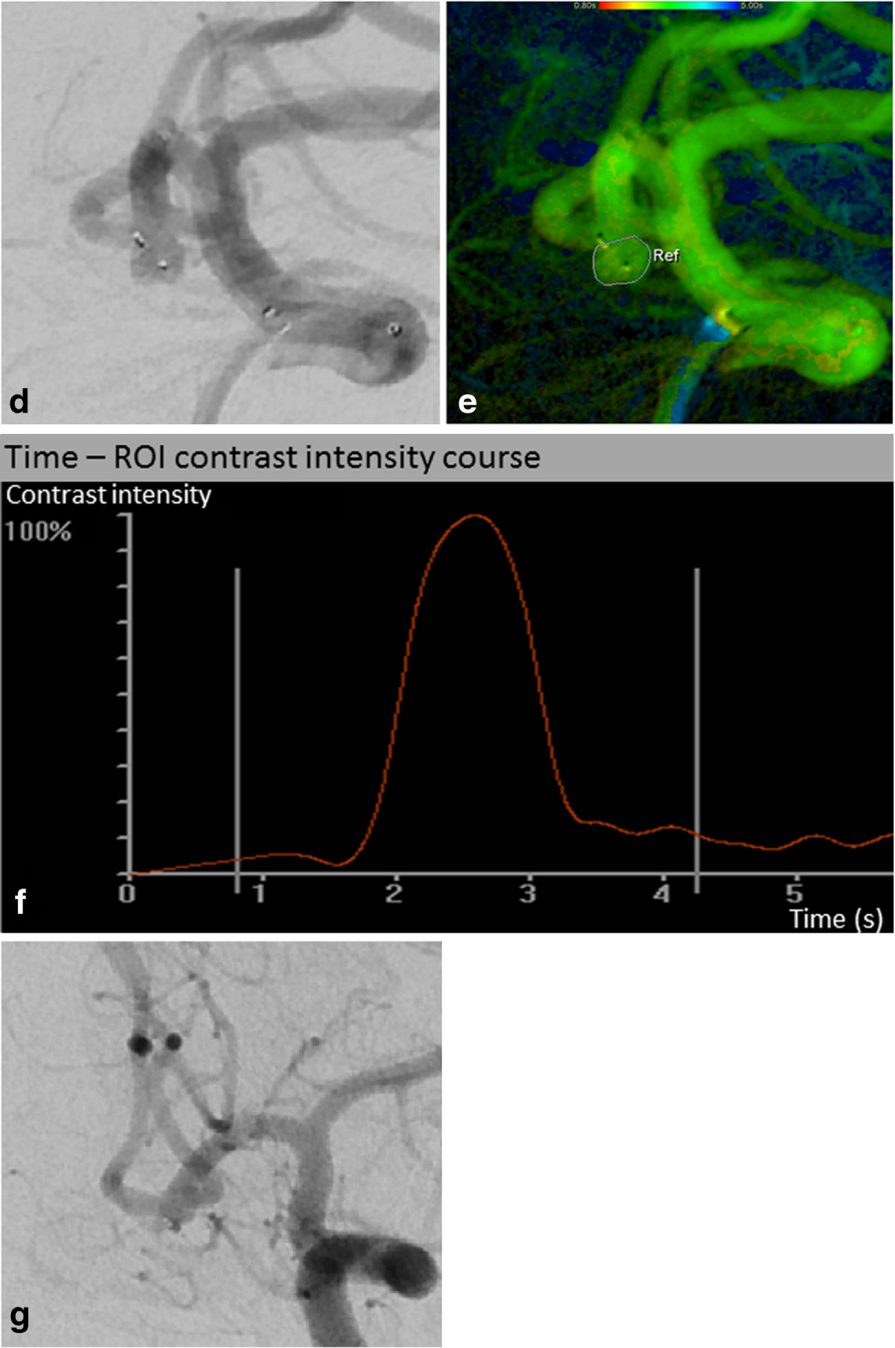
DSA presents a broad-based aneurysm at the anterior communicating artery (**a**). After parametric color coding, a ROI is drawn around the perimeter of the aneurysm (**b**) and aneurysmal TDC is generated (**c**). The TDC is quantified by calculating rTTP of aneurysmal filling, aneurysmal wash-in/inflow velocity, and aneurysmal wash-out/outflow velocity. For illustration, these parameters are blue-marked in the graph. DSA shows the aneurysm directly after WEB deployment (**d**). Identically to preinterventional, after parametric color coding a ROI is drawn around the perimeter of the aneurysm (**e**) and aneurysmal TDC generated (**f**). TDC analysis reveals that rTTP of aneurysmal filling and aneurysmal wash-in/inflow velocity are unchanged, but aneurysmal wash-out/outflow velocity is reduced by 58%. This hemodynamic change might be considered beneficial concerning aneurysm occlusion since our supposed threshold of 32% postinterventional outflow reduction is exceeded. Accordingly, DSA follow-up after 6 months presents only opacification of the proximal recess of WEB (grade 0′ according to the BOSS classification), rated as complete occlusion (**g**)

**Fig. 2:**
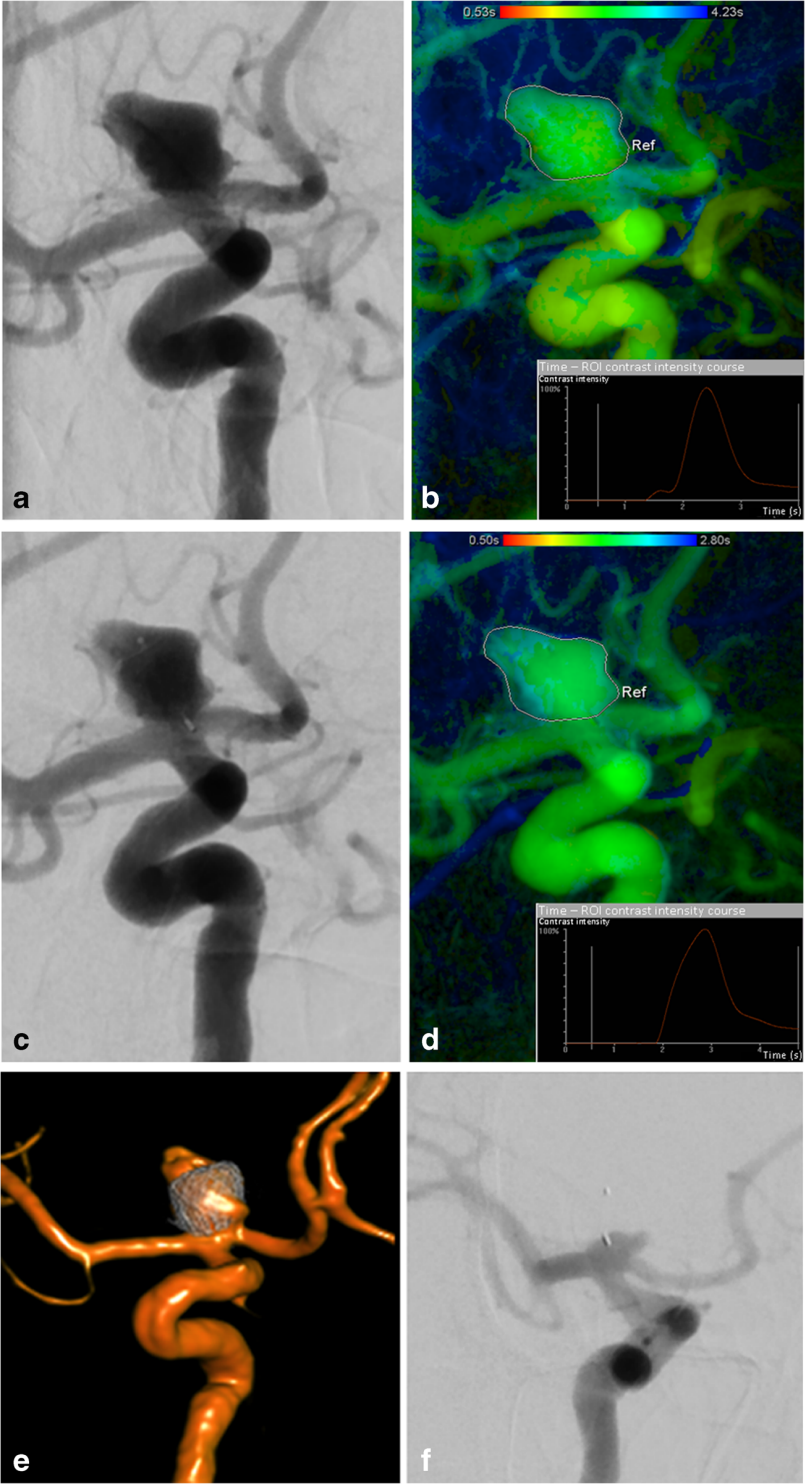
DSA shows a broad-based aneurysm at carotid terminus before (**a**) and after (**c**) treatment with WEB. At (**b**) and (**d**), the corresponding pre- and postinterventional TDC analysis is presented. It reveals that after WEB implanting rTTP of aneurysmal filling is prolonged by 63%, aneurysmal wash-in/inflow velocity is reduced by 38%, and aneurysmal wash-out/outflow velocity is decreased by 31%. 3D rotational run directly after WEB detachment confirms correct positioning of the device (**e**). DSA follow-up after 6 months demonstrates aneurysm remnant (grade 3 according to the BOSS classification) (**f**) that was re-treated

The target pre- and postinterventional 2D DSA series were postprocessed using commercially available iFlow software (syngo iFlow, Siemens AG, Healthineers) that allows an automated conversion of the DSA series into color-coded images. In addition, the acquisition of TDCs in freely chosen regions of interest (ROIs) is possible. Each 2D DSA series acquired immediately after WEB detachment was used for analysis as postinterventional series. First, an arterial TDC distal to the tip of the guiding catheter was generated pre- and postinterventionally and compared, confirming in this way the robustness of the manual contrast injections. After that, in each series, a ROI was manually drawn around the perimeter of the aneurysm and aneurysmal TDC was generated representing the intraaneurysmal dynamic behavior of the mixture of blood and contrast material (compare Figs. 1 and 2). The TDC was quantified by calculating the following parameters:

- Relative time-to-peak (rTTP) of aneurysmal filling: time from nadir of the curve to maximum peak, measured in seconds (s)
- Aneurysmal inflow velocity/wash-in: average slope of the curve before peak, measured in “density change per s”
- Aneurysmal outflow velocity/wash-out: average slope of the curve after peak, measured in “density change per s”

Based on the electronically gained coordinates of the TDC, the velocity is calculated by curve arithmetic: velocity = ∆*y* /∆*x*.

In addition, mean aneurysmal flow velocity was calculated to assess the degree of aneurysmal hemodynamic stress: mean flow velocity = (aneurysmal inflow + outflow velocity)/2.

Moreover, the angle between parent artery and surface of the WEB was measured.

Statistical analysis was performed using the software SPSS statistics. Mean values and standard deviation before and after treatment as well as post-/pre-ratios were calculated for each parameter. The differences between the pre- and postinterventional values were tested for significance using a Wilcoxon signed-rank test. A level of *p* ≤ 0.05 was considered statistically significant. To assess differences between the occluded and the non-occluded group, the pre-/postinterventional differences and the post-/pre-ratios of each parameter were tested for significance using the Mann-Whitney *U* test. Additionally, the sample group was dichotomized into aneurysms located within the anterior and the posterior circulation and subgroup analysis was performed. Logistic regression was used to identify independent parameters which might significantly predict aneurysm occlusion.

## Results

Immediately after WEB implanting, mean rTTP of aneurysmal filling demonstrated a highly significant prolongation by 52% (*p* = 0.001) (compare Table 1). In addition, aneurysmal inflow and outflow velocity as well as mean flow velocity showed a highly significant decrease after intervention (*p* < 0.001). While mean aneurysmal outflow velocity was reduced by 49% after WEB implanting, mean inflow velocity was reduced by 33% only. The mean angle between parent artery and WEB surface was 95° (± 18°). In only three cases, minimal protrusion of the WEB into parent artery by 0.4 mm, 0.7 mm, and 0.8 mm was observed, meaning a relative protrusion of 21%, 28%, and 27% in relation to the vessel diameter, respectively.

**Table 1:** Pre- and postinterventional aneurysmal flow parameters

	Inflow pre (density change/s)	Inflow post (density change/s)	Outflow pre (density change/s)	Outflow post (density change/s)	rTTP pre (s)	rTTP post (s)	Mean flow pre (density change/s)	Mean flow post (density change/s)
Mean value	12.7	9.3	8.6	3.7	0.8	1.1	6.24	3.44
Standard deviation	4.6	3.6	6.0	2.4	0.4	0.5	1.87	1.29
Difference pre- vs. post-value	*p* < 0.001		*p* < 0.001		*p* = 0.001		*p* < 0.001	

Neither the pre-/postinterventional differences nor the post/pre-ratios of the flow parameters nor the WEB angulation showed a statistically significant difference between the occluded and the non-occluded group (all *p* values > 0.05). Subgroup analysis demonstrated no significant difference of the flow parameters between the ruptured and the unruptured group as well as between aneurysms located within the anterior and the posterior circulation. In logistic regression analysis, no single flow parameter reached significance level concerning predicting aneurysm occlusion.

Eighteen of the forty aneurysms (45%) showed complete occlusion at angiographic follow-up after 6 months. One unruptured aneurysm in an elderly patient (73 years) presented delayed rupture 3 h after WEB deployment. In this case, aneurysmal outflow velocity was reduced by 48%, but inflow velocity showed only minimal decrease by 4% and rTTP of aneurysmal filling was only slightly prolonged by 4%. Concomitantly, the magnitude of the postinterventional mean flow velocity was high (5.06 density change/s). A comparable flow constellation after WEB implanting could be revealed in only one other case of our sample group in a young patient (40 years). Additionally, in the case of delayed aneurysm rupture, only incomplete covering of the whole aneurysm with the WEB was possible because of an irregular aneurysm shape.

If a postinterventional reduction of at least 32% of the outflow velocity was set as cut-off, complete aneurysm occlusion after 6 months was predictable with high sensitivity of 83% but only low specificity of 56%. If a postinterventional reduction of at least 39% of the inflow velocity was added as an additional parameter, sensitivity raised up to 89% but at the cost of a low specificity of 39%. If the postinterventional outflow was reduced only up to 3%, incomplete aneurysm occlusion at follow-up was predictable with 100% sensitivity and specificity, but this observation is based on only two cases.

## Discussion

Flow disruption by WEB implanting has emerged as an innovative endovascular concept for aneurysm treatment [3–8], but quantifiable data concerning the hemodynamic effect of this treatment strategy are lacking. In our study, we investigated in vivo hemodynamic changes following WEB deployment using TDC analysis.

Our hemodynamic analysis reveals that by WEB implanting the mean rTTP of aneurysmal filling is increased significantly. In addition, WEB treatment alters highly significantly the intraaneurysmal flow velocity reducing more the outflow than the inflow velocity. The aneurysmal hemodynamic stress is also significantly reduced by WEB deployment. The level of the hemodynamic changes is comparable to that reported for intraluminal flow-diverting stents. Here, a mean reduction of 51% for aneurysmal outflow velocity and of 37% for aneurysmal inflow velocity was observed [17]. These data suggest that the hemodynamic effect of intrasaccular flow disruption and intraluminal flow diversion seems comparable.

The midterm rate of 45% of complete aneurysm occlusion after WEB treatment in our study is in line with the data provided by a meta-analysis reporting a 39% complete occlusion rate [5]. In our study, no flow parameter turned out as independent factor having significant predictive value concerning complete aneurysm occlusion but this might be possible in a larger cohort. Based on our preliminary results, reducing the aneurysmal outflow velocity by WEB might be crucial concerning complete aneurysm occlusion. A suitable threshold value might range about 32% of postinterventional outflow reduction, providing in our series high sensitivity (83%) at low specificity (56%) concerning the prediction of aneurysm occlusion. The level of this possible threshold value is also similar to that described for flow-diverting stents. Here, a 35% reduction of aneurysmal outflow velocity seems to be a predictive factor concerning aneurysm occlusion [17]. Moreover, a nearly unchanged postinterventional aneurysmal outflow might be considered a prognostically unfavorable factor concerning complete aneurysm occlusion and change of the treatment regime should be considered.

The similar hemodynamic effect of WEB and flow-diverting stents implies that WEB treatment harbors potentially the similar risk profile including aneurysm rupture as reported for flow-diverting stents. Moreover, if intraaneurysmal flow alteration and aneurysm thrombosing are considered similar, it means in consequence that after WEB deployment aneurysm closure can be delayed by weeks or months. Additionally, our flow analysis reveals that in some aneurysms intraaneurysmal flow is only slightly changed by WEB implanting. This is of special concern in ruptured aneurysms, and in principle it could mean that some aneurysms, especially acutely ruptured, might not be adequately protected in the short term due to persisting aneurysmal wall stress. This hypothesis might be a possible explanation for the re-rupture of WEB-treated aneurysms [16, 18] or the occurrence of delayed ipsilateral parenchymal hemorrhage [19]. This hypothesis is further substantiated from our data with one case of delayed rupture of an unruptured aneurysm after WEB deployment. In this case, aneurysmal inflow velocity showed only minimal decrease while the outflow velocity was reduced substantially and the postinterventional aneurysmal hemodynamic stress remained on a high level. Based on this limited single observation, only mild reduction of aneurysmal inflow velocity by WEB at concomitantly remaining high aneurysmal hemodynamic stress might be a potentially critical flow constellation or co-factor concerning delayed aneurysm rupture due to persisting aneurysmal wall stress and endothelial injury. Additionally, incomplete covering of the whole aneurysm with the WEB might be also a potentially critical co-factor. Thus, WEB users should be aware of its hemodynamic functioning and the possible implicit risk potential. From this point of view, it seems worthy to discuss whether for aneurysms for whom various treatment options exist WEB deployment should be the preferable approach.

Computational fluid dynamics (CFD) simulations before implantation of an intrasaccular flow diverter suggested an association between the aneurysm inflow ratio and compression of the intrasaccular device [20]. Recently developed methods for CFD modeling of hemodynamics in aneurysms treated with intrasaccular devices showed also a distinct postinterventional reduction of aneurysm flow velocities [21]. However, CFD simulations are limited by simplifications of hemodynamic features and lack of patient-specific information on physiological flow velocity. In contrast to CFD simulations, in vivo evaluation using time-density analysis is less time consuming. As an easy-to-handle technique, it could be used for real-time flow analysis during intervention before WEB detachment. Thus, the hemodynamic efficiency of WEB could be monitored directly and maybe favorable or adverse flow modulations could be identified. Therefore, intraprocedural flow analysis before WEB detachment could be included additively into the decision-making process on the most appropriate WEB and, in case of an unfavorable flow result, removal of the WEB for a different one or changing the endovascular approach might be considered. In this way, flow analysis might contribute to patient safety and improving long-term results.

Our study results are certainly limited by the small sample size. Moreover, manual and not mechanical injections were used for DSA acquisition. Manual injections may vary minimally but have the advantage of reduced hemodynamic disturbance during the injection period compared with injections using a power injector. Recently published data demonstrate that relatively low rate hand injections may be superior to mechanical injections and best-suited for aneurysmal TDC analysis since consistency in the angiograms is the most relevant parameter concerning TDC analysis [22]. Furthermore, aneurysm occlusion has to be considered multifactorial and other than sole hemodynamic factors which may not be predicted by flow characteristics alone, i.e., concomitant antiplatelet therapy, influence aneurysm thrombosis. Further validation of TDC analysis in a larger patient cohort seems mandatory to substantiate our preliminary results.

## Conclusion

In vivo flow quantification reveals that WEB implanting has a highly significant flow-disrupting effect on aneurysmal flow parameters, inducing rTTP prolongation of aneurysmal filling as well as reduction of aneurysmal outflow and, to a lesser extent, inflow velocity. The aneurysmal hemodynamic stress is also significantly decreased by treatment. Based on our preliminary results, reducing the aneurysmal outflow velocity might be crucial concerning aneurysm occlusion, but in our small cohort no flow parameter reached significance level as independent factor. The hemodynamic effect of the WEB is on comparable level to flow-diverting stents meaning in consequence that aneurysm closure can be delayed, and in case of only slight inflow changes at concomitantly remaining high aneurysmal hemodynamic stress, some aneurysms might not be adequately protected in the short term due to persisting endothelial injury. Thus, WEB users should be aware of its hemodynamic functioning and its possible implicit risk potential.

## Abbreviations

BOSS: Beaujon Occlusion Scale Score
CFD: Computational fluid dynamics
DSA: Digital subtraction angiography
FDS: Flow-diverting stent
ICA: Internal carotid artery
rTTP: Relative time-to-peak
TDC: Time-density curve
WEB: Woven EndoBridge
VA: vertebral artery

## References

[1] Klisch J, Sychra V, Strasilla C, Liebig T, Fiorella D (2011). The Woven EndoBridge cerebral aneurysm embolization device (WEB II): initial clinical experience. Neuroradiology.

[2] Pierot L, Moret J, Turjman F, Herbreteau D, Raoult H, Barreau X, Velasco S, Desal H, Januel AC, Courtheoux P, Gauvrit JY, Cognard C, Soize S, Molyneux A, Spelle L (2015). WEB treatment of intracranial aneurysms: feasibility, complications, and 1-month safety results with the WEB DL and WEB SL/SLS in the French Observatory. AJNR Am J Neuroradiol.

[3] Pierot L, Moret J, Barreau X, Szikora I, Herbreteau D, Turjman F, Holtmannspötter M, Januel AC, Costalat V, Fiehler J, Klisch J, Gauvrit JY, Weber W, Desal H, Velasco S, Liebig T, Stockx L, Berkefeld J, Molyneux A, Byrne J, Spelle L (2018). Safety and efficacy of aneurysm treatment with WEB in the cumulative population of three prospective, multicenter series. J Neurointerv Surg.

[4] Lv X, Zhang Y, Jiang W (2018). Systematic review of Woven EndoBridge for wide-necked bifurcation aneurysms: complications, adequate occlusion rate, morbidity, and mortality. World Neurosurg.

[5] Asnafi S, Rouchaud A, Pierot L, Brinjikji W, Murad MH, Kallmes DF (2016). Efficacy and safety of the Woven EndoBridge (WEB) device for the treatment of intracranial aneurysms: a systematic review and meta-analysis. AJNR Am J Neuroradiol.

[6] Goertz L, Liebig T, Siebert E et al (2019) Extending the indication of Woven EndoBridge (WEB) embolization to internal carotid artery aneurysms: a multicenter safety and feasibility study. World Neurosurg. 10.1016/j.wneu.2019.02.198 10.1016/j.wneu.2019.02.198 30876989

[7] Pierot L, Gubucz I, Buhk JH, Holtmannspötter M, Herbreteau D, Stockx L, Spelle L, Berkefeld J, Januel AC, Molyneux A, Byrne JV, Fiehler J, Szikora I, Barreau X (2017). Safety and efficacy of aneurysm treatment with the WEB: results of the WEBCAST 2 study. AJNR Am J Neuroradiol.

[8] Pierot L, Moret J, Turjman F, Herbreteau D, Raoult H, Barreau X, Velasco S, Desal H, Januel AC, Courtheoux P, Gauvrit JY, Cognard C, Molyneux A, Byrne J, Spelle L (2016). WEB treatment of intracranial aneurysms: clinical and anatomic results in the French observatory. AJNR Am J Neuroradiol.

[9] Papagiannaki C, Spelle L, Januel AC, Benaissa A, Gauvrit JY, Costalat V, Desal H, Turjman F, Velasco S, Barreau X, Courtheoux P, Cognard C, Herbreteau D, Moret J, Pierot L (2014). WEB intrasaccular flow disruptor-prospective, multicenter experience in 83 patients with 85 aneurysms. AJNR Am J Neuroradiol.

[10] Ding YH, Lewis DA, Kadirvel R, Dai D, Kallmes DF (2011). The Woven EndoBridge: a new aneurysm occlusion device. AJNR Am J Neuroradiol.

[11] Roy D, Milot G, Raymond J (2001). Endovascular treatment of unruptured aneurysms. Stroke.

[12] Lawson A, Goddard T, Ross S, Tyagi A, Deniz K, Patankar T (2017). Endovascular treatment of cerebral aneurysms using the Woven EndoBridge technique in a single center: preliminary results. J Neurosurg.

[13] Fiorella D, Arthur A, Byrne J, Pierot L, Molyneux A, Duckwiler G, McCarthy T, Strother C (2015). Interobserver variability in the assessment of aneurysm occlusion with the WEB aneurysm embolization system. J Neurointerv Surg.

[14] Cognard C, Januel AC (2015). Remnants and recurrences after the use of the WEB intrasaccular device in large-neck bifurcation aneurysms. Neurosurgery.

[15] Caroff J, Mihalea C, Ikka L, Moret J, Spelle L (2015). Interobserver variability in the assessment of aneurysm occlusion with the WEB aneurysm embolisation system. J Neurointerv Surg.

[16] Caroff J, Mihalea C, Klisch J, Strasilla C, Berlis A, Patankar T, Weber W, Behme D, Jacobsen EA, Liebig T, Prothmann S, Cognard C, Finkenzeller T, Moret J, Spelle L (2015). Single-layer WEBs: intrasaccular flow disrupters for aneurysm treatment—feasibility results from a European study. AJNR Am J Neuroradiol.

[17] Gölitz P, Struffert T, Rösch J, Ganslandt O, Knossalla F, Doerfler A (2015). Cerebral aneurysm treatment using flow-diverting stents: in-vivo visualization of flow alterations by parametric colour coding to predict aneurysmal occlusion: preliminary results. Eur Radiol.

[18] Clajus C, Strasilla C, Fiebig T, Sychra V, Fiorella D, Klisch J (2017). Initial and mid-term results from 108 consecutive patients with cerebral aneurysms treated with the WEB device. J Neurointerv Surg.

[19] Arthur AS, Molyneux A, Coon AL, Saatci I, Szikora I, Baltacioglu F, Sultan A, Hoit D, Delgado Almandoz JE, Elijovich L, Cekirge S, Byrne JV, Fiorella D, WEB-IT Study investigators (2019). The safety and effectiveness of the Woven EndoBridge (WEB) system for the treatment of wide-necked bifurcation aneurysms: final 12-month results of the pivotal WEB Intrasaccular Therapy (WEB-IT) Study. J Neurointerv Surg.

[20] Caroff J, Mihalea C, Da Ros V (2017). A computational fluid dynamics (CFD) study of WEB-treated aneurysms: can CFD predict WEB “compression” during follow-up?. J Neuroradiol.

[21] Mut F, Chung BJ, Chudyk J (2019). Image-based modeling of blood flow in cerebral aneurysms treated with intrasaccular flow diverting devices. Int J Numer Methods Biomed Eng.

[22] Sadasivan C, Dholakia R, Peeling L et al (2019) Angiographic assessment of the efficacy of flow diverter treatment for cerebral aneurysms. Interv Neuroradiol. 10.1177/1591019919860829 10.1177/1591019919860829 PMC6838848 31296064

